# Abstracts of Notes on the Treatment of Exophthalmic Goitre

**Published:** 1893-11

**Authors:** J. Madison Taylor

**Affiliations:** Philadelphia; Neurologist to the Howard Hospital; Assistant Physician to the Infirmary for Nervous Diseases; Professor of the Diseases of Children, Philadelphia Polyclinic


### ABSTRACTS OF NOTES ON THE TREATMENT OF EXOPHTHALMIC GOITRE.*

By J. MADISON TAYLOR, M. D.,

Neurologist to the Howard Hospital; Assistant Physician to the Infirmary for Nervous Diseases; Professor of the Diseases of Children, Philadelphia Polyclinic.

Dr. Taylor takes up the subject of exophthalmic goitre as understood at the present time, reviews its relationship to other vascular disturbances, associated with enlargement of the thyroid, as myxœdema, cretinism, and acromegalia. He does not see that at present the thyroid physiology warrants any large measures of hope that we shall treat this malady through thyroid juices, as in the case of the other disorders mentioned, and yet promises to explore this field later.

He confines himself chiefly to the analysis of a number of cases long under his care, some of them for many years, and most of whom are well, summarizing the means through which they were improved. The treatment of maladies which demand but little constant supervision, and yet require prolonged watchfulness, is more or less difficult and unsatisfactory because such cases wander away, will not persist in treatment, and from their nature of unhopefulness, have a tendency to fall into the hands of other medical men and have their treatment changed; also the exigencies of labor, fatigue, changed surroundings, etc., make it difficult to pursue any consistent plan, even with those inclined to be faithful. He takes the ground that this disorder is capable of much more hopeful treatment than is generally thought; that most cases are enormously improved, if rightly handled; that many get well.

The question of treatment is chiefly considered from the standpoint of his own experience. This consists in:

*Paper read before the American Medical Association, Milwaukee.

(1) Regulated rest, with carefully graduated activities as time and circumstances warrant, systematic measures directed to the upbuilding or the general health, careful attention to nutrition, recognizing the trophic elements in the disorder.

(2) Careful attention to vascular conditions, which are most noticeably at fault and which demand the most constant treatment. This consists of varying measures, regulating vaso-motor activities, as well as loss of nervous force through the easily disturbed nervous balance. This is done through attention to the skin from the surface by means of carefully regulated measures from without, and next the review of certain remedies which seem to exert a control over the constant tendency to vaso-motor neuroses, both superficial and deep.

(3) To search out constitutional defects and remedy these, eliminate accidental poisons, either diathetic or temporary, eliminate toxines, as of internal sewage poisoning, which has a very direct bearing.

(4) The regulation of the nervous or emotional balance by careful attention to the habits and environment of the individual, carefully regulating, so far as possible, the habitual emotional strains and the possible ones, making much of moral teachings to those whose will power and mental equilibrium are gravely at fault constitutionally and as inevitable entanglements due to months or years of suffering and susceptibilities.

(5) A careful consideration of such measures as are regarded as specific, as electricity, of which much has been made by many writers, but whose value consists chiefly in the system and encouragement which is thereby exerted over the disorded emotional states.

Finally, a summary of the cases under observation when the original paper was written in 1888 and the report of a number of others since then, and a careful review of the symptomatology, and various means of treatment recently found to be of value.

